# Excessive Maternal Weight and Diabetes Are Risk Factors for Macrosomia: A Cross-Sectional Study of 42,663 Pregnancies in Uruguay

**DOI:** 10.3389/fendo.2020.588443

**Published:** 2020-11-03

**Authors:** Jimena Pereda, Isabel Bove, Maria M. Pineyro

**Affiliations:** ^1^ Clinica de Endocrinología y Metabolismo, Hospital de Clínicas, Facultad de Medicina, Universidad de la República, Montevideo, Uruguay; ^2^ Departamento de Ciencias Cognitivas y de la Salud, Universidad Católica del Uruguay, Montevideo, Uruguay

**Keywords:** overweight, obesity, pregnancy, gestational diabetes, pregestational diabetes, macrosomia

## Abstract

**Objective:**

To evaluate the risk of macrosomia in newborns from women with gestational diabetes, pregestational diabetes, overweight, and obesity in Uruguay in 2012, as well as its association with prolonged pregnancy, maternal age, multiparity, and excessive gestational weight gain (EGWG).

**Methods:**

We performed a cross-sectional study of 42,663 pregnant women. The risk of macrosomia was studied using logistic regression.

**Results:**

Mean maternal age was 26.7 ± 6.8 years. Pregestational overweight and obesity was present in 20.9% and 10.7% of women, respectively. There were 28.1% and 19.8% of women overweight and obese at the end of the pregnancy, respectively. Furthermore, 0.5% had pregestational diabetes and 8.5% were multiparous. Twenty two percent developed gestational diabetes and 44.9% had EGWG. The prevalence of macrosomia was 7.9%, significantly more prevalent in males (10.0% vs. 5.5%, p<0.005). Univariate analysis showed that obesity and overweight pre-pregnancy, obesity and overweight at the end of pregnancy, EGWG, pregestational diabetes, gestational diabetes, multiparity, prolonged pregnancy, and male newborn were strongly associated with macrosomia (p<0.0001). Maternal age >35 years did not increase the risk of macrosomia. After multiple logistic regression macrosomia was more likely in pre-gestational obese women (OR 1.24; CI 1.07–1.44), overweight women at the end of pregnancy (OR 1.66; CI 1.46–1.87), obese women at the end of pregnancy (OR 2.21; CI 1.90–2.58), women with EGWG (OR 1.78; CI 1.59–1.98), pregestational diabetes (OR 1.75; CI 1.15–2.69), gestational diabetes (OR 1.39; CI 1.25–1.53), prolonged pregnancy (OR 2.67; CI 2.28–3.12), multiparity (OR 1.24; CI 1.04–1.48), and male newborn (OR 1.89; CI 1.72–2.08).

**Conclusion:**

Maternal overweight, obesity, EGWG, and gestational diabetes are prevalent in Uruguay, increasing the risk of macrosomia. Efforts to implement strategies to decrease the prevalence of overweight and obesity among women of reproductive age are essential to improve maternal and neonatal outcomes.

## Introduction

Fetal macrosomia is defined as neonate birthweight ≥4,000 g and large for gestational age as birthweight above the 90^th^ centile. Worldwide prevalence of fetal macrosomia is approximately 9%, with broad variations between countries (1). In Uruguay it is reported in 6% to 11.1% of newborns (2, 3).

Macrosomia is associated with maternal and fetal complications such as preterm birth, postpartum hemorrhage, maternal birth canal trauma, a higher risk of cesarean delivery, shoulder dystocia, fetal asphyxia, and neonatal hypoglycemia (4–6). In addition, it increases the risk of developing obesity, insulin resistance, metabolic syndrome, and cancer later in life (7–9).

Maternal overweight, excessive gestational weight gain and gestational diabetes are known risk factors for macrosomia. The prevalence of obesity and gestational diabetes is increasing worldwide. In the United States between 18.5% and 38.3% of women are obese at conception or delivery (10, 11).

In Uruguay, a survey of 900 randomly selected adults reported 53% of women with body mass index (BMI) ≥25 kg/m^2^. Fourteen percent of women between 18 and 35 years were overweight, and 8% obese. Moreover, 33% of women between 36 and 45 years were overweight and 22% obese (12).

Diabetes affects 6%–9% of pregnancies, the majority (99%) having gestational diabetes (13, 14). Moreover, with the new diagnostic criteria for diabetes in pregnancy adopted by the International Association of Diabetes and Pregnancy Study Groups (IADPSG), World Health Organization (WHO), and American Diabetes Association (ADA) the prevalence of hyperglycemia in pregnancy has increased to an estimated of 17% (15). This new diagnostic criteria was based on The Study of Hyperglycemia and Adverse Pregnancy Outcomes (HAPO) which demonstrated a linear continuous correlation between levels of maternal blood glucose and adverse perinatal outcomes (16).

Maternal overweight and obesity regularly coexist with gestational diabetes. Obesity and gestational diabetes are recognized risk factors for adverse pregnancy outcomes such as preterm delivery, hypertension, primary cesarean delivery, and large for gestational age fetuses and macrosomia (17, 18).

Infants of women with obesity or gestational diabetes are at an increased risk of becoming overweight or obese at a young age and are more likely to develop type 2 diabetes, metabolic syndrome and cancer later in life (19, 20). In a cohort of 23832 Uruguayan women who delivered babies from 2010 to 2012 there were 23.6% and 9.4% women overweight and obese pre-pregnancy, respectively. In addition, high pre-gestational BMI and EGWG were significantly associated with macrosomia in this cohort.

The aim of this study was to evaluate the risk of macrosomia in newborns from women with gestational diabetes (GD) and overweight/obesity in Uruguay in 2012, as well as it association with prolonged pregnancy, maternal age and multiparity. In addition, we evaluated prevalence and risk of macrosomia in newborns from mothers with pregestational diabetes (PGD) and from those with EGWG.

## Materials and Methods

We performed an observational and cross-sectional study of 42, 663 pregnant women and their newborns in Uruguay in 2012. There where 45,790 SIP valid forms form pregnant women, with 3157 (6.9%) cases meeting exclusion criteria. The data was collected from the Perinatal Information System (SIP) records, which are managed by the Latin American Center for Perinatology, Women and Reproductive Health (CLAP), a unit from the WHO. The SIP was established in 2000 and its use became mandatory in 2009. A structured form, which includes exhaustive data of the pregnancy and delivery as well as perinatal health of the newborn, is completed by the gynecologist during pregnancy and by the delivery hospital. Data form the SIP of all maternity hospitals of the country was analyzed.

Exclusion criteria included abortion, stillbirth, multiple pregnancy, gestation less than 22 weeks, and extreme values in maternal weight (pre-pregnancy weight or at end of gestation less than 35 kg or more than 200 kg or BMI less than 14 kg/m^2^ or more than 60 kg/m^2^). In addition, extreme values in neonatal birth weight where excluded (less than 600 gr or more than 6.500 g), as well as those records without data birth weight.

Pre-pregnancy BMI (kg/m2) was defined as follows: <18.5 = underweight; 18.5–24.9 = normal weight; 25–29.9 = overweight; 30 or higher = obesity.

Gestational weight gain was calculated as the difference between weight at delivery and pre-pregnancy self-reported weight. Excessive gestational weight gain was considered when it exceeded the total weight gain recommendations from the Institute of Medicine (IOM). Gestational weight gain according to the IOM consists of: 1) underweight: 12.5–18.0 kg; 2) normal weight: 11.5–16.0 kg; 3) overweight: 7.0–11.5 kg; and 4) obese: 5.0–9.0 kg. Body mass index at the end of gestation was calculated with last recorded pregnancy weight available in the last follow up, defined as the prenatal visit within 2 weeks of delivery. Categories of nutritional status where defined relying on a modified chart by Atalah et al. based on longitudinal studies in Chile and adopted by Latin American countries (21). Categories based on BMI at the end of pregnancy consists of: 1) normal weight: 25.00–29.00; 2) overweight: 29.10–33.10; and 3) obese: >33.10.

Diabetes was categorized as gestational diabetes according to IADPSG criteria of fasting glucose ≥92 mg/dl, as information on 2-h glucose after an oral glucose tolerance test (OGTT) is not available in the SIP records. Fasting glucose levels are recorded before 20 weeks and after 20 weeks of gestation in the SIP form. Older maternal age was defined as women aged 35 years or older. Multiparity was considered when women had three or more live births. Prolonged pregnancy was defined as the one that persisted beyond 41 weeks of gestation. Macrosomia was defined as birth weight equal or above 4000 grams.

The association of qualitative variables was performed by Pearson´s Chi-square test and the risk of macrosomia was studied using a model of binary logistic regression, expressed as odds ratios (OR) with 95% confidence intervals (95% CI). The tests were considered statistically significant at an alpha level (α) of 0,05 (p<0,05). Multiple regression analysis was done to estimate the relative contribution of independent variables found to be significant in univariate analysis (overweight and obesity pre-pregnancy and at the end pregnancy, EGWG, pregestational and gestational diabetes, multiparity, prolonged pregnancy, and male newborn) to macrosomia (adjusted ORs). We examined potential collinearity using variance inflation factors (VIF) and tolerance test (TT). Results indicated very low levels of multicollinearity with a maximum VIF at <2.2 and TT>0.2, expect for normal weight at the end of pregnancy so it was excluded from multiple regression analysis (it provided redundant information). In addition, we performed forward and backward stepwise regression, with the variables without a statistical relationship progressively eliminated. Both forward and backward selections yield the same results.

Statistical analysis was performed with SPSS version 17.0. The de-identified SIP data was provided by the Uruguayan Ministry of Public Health; its analysis was not subject to human subjects review.

## Results

We evaluated 42,663 singleton deliveries in 2012 in Uruguay. Mean maternal age was 26.7 ± 6.8 years. Population demographics are shown in **Table 1**.

**Table 1:** Population demographics.

Variable	Total (n = 42,663)
Age (y)	26.7 ± 6.8
Age >35 (y)	4629 (10.9)
BMI pre-pregnancy	24.0 ± 4.8
BMI at end of pregnancy	29.1 ± 4.9
Weight gain (kg)	14.3 ± 6.9
Prolonged pregnancy	2086 (4.9)
Multiparous	3634 (8.5)
Diabetes (DM)	
Pre-gestational	304 (0.7)
Gestational	9252 (22)
Pre-pregnancy weight	
Overweight	7667 (20.9)
Obese	3965 (10.7)
Weight at end of pregnancy	
Overweight	9860 (28.1)
Obese	6951 (19.8)
Excessive gestational weight gain	11422 (44.9)
Gestational age (weeks)	38.5 ± 1.9
Male newborn	21802 (51.2)

Data are n (%), mean ± SD.

Pre-gestational overweight and obesity was recorded in 20.9% and 10.7% of women, respectively. There were 28.1% and 19.8% of women overweight and obese at the end of the pregnancy, respectively. Twenty-three percent of women (n=5219) with normal weight at the beginning of the pregnancy were overweight at the end it, as well as 34.4% (n=2638) of those overweight pre-pregnancy ended up being obese over the course of it. Excessive gestational weight gain was more prevalent in women being overweight or obese before pregnancy (**Table 2**).

**Table 2:** Population demographics according to pre-pregnancy weight.

Variable	Normal weight (n = 22588)	Over weight ( n =7667)	Obese (n = 3965)
Age (years)	26.4 ± 6.7 (12–48)	27.9 ± 6.6 (12–50)	28.8 ± 6.4 (14–48)
BMI	21.8 ± 1.7	27.1 ± 1.4	34.3 ± 4.3
Excessive gestational weight gain	5937 (26.3)	3339 (44.3)	1560 (39.3)
Gestational age of newborn (weeks)	38.5 ± 1.8 (23–42)	38.6 ± 1.8 (22–42)	38.4 ± 2.0 (23–42)
Birth weight (grams)	3259.8 ± 538.9 (670–5290)	3361.2 ± 565.5 (675–6000)	3404.0 ± 628.6 (700–5970)
Birth weight (grams) percentiles			
P50	3290	3400	3450
P75	3600	3710	3795
P90	3880	4010	4125
Macrosomia	1458 (6.5)	788(10.3)	593 (15.0)
Male newborn	11513 (51.0)	3923 (51.2)	2048 (51.7)

Data are n (%), mean ± SD (range).

Furthermore, 0.5% had PGD, 22% developed GD, and almost half had EGWG. Gestational diabetes was present in one third of women with obesity before pregnancy, and in 18% of those without it.

**Figure 1:**
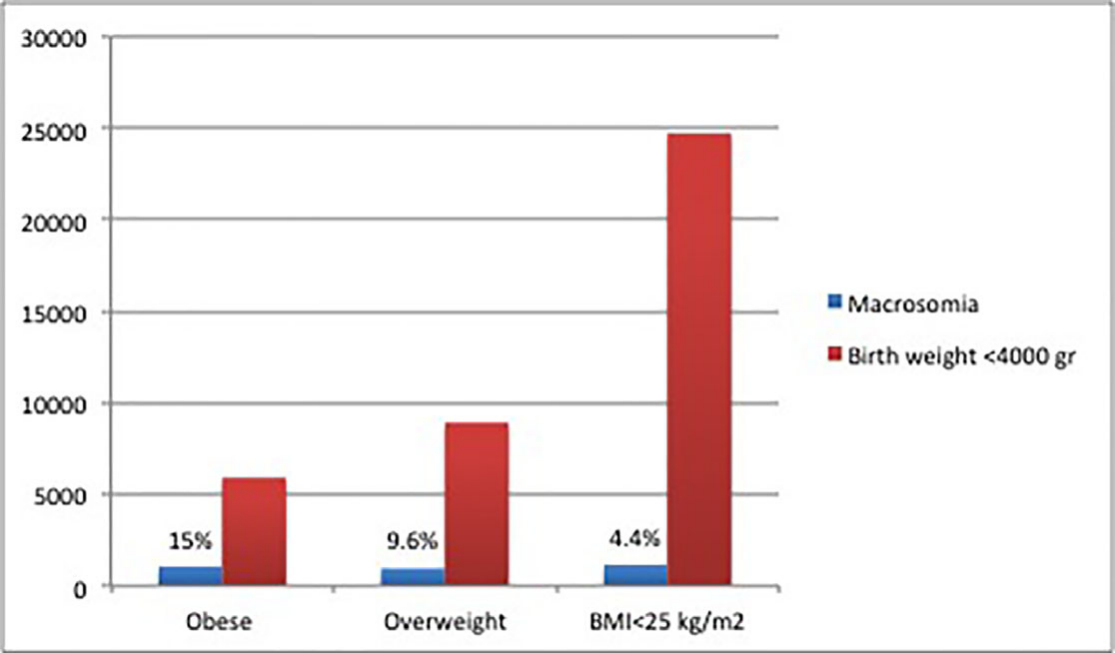
Prevalence of macrosomia related to birth weight at the end of pregnancy.

The prevalence of macrosomia was 7.9%, significantly more prevalent in males (10.0% vs. 5.5%, p<0.005). Macrosomia was more prevalent in obese women at the end of pregnancy (**Figure 1**).

**Table 3** shows frequency of macrosomia related to clinical factors. Univariate analysis showed that obesity and overweight pre-pregnancy, obesity, and overweight at the end of pregnancy, EGWG, PGD, GD, multiparity, prolonged pregnancy, and male newborn were strongly associated with macrosomia (p<0.0001). Maternal age >35 years did not increase the risk of macrosomia (**Table 3**).

**Table 3:** Incidence of primary outcome according to clinical factors.

Variable	Total N	No macrosomia		Macrosomia N%		Crude	p
	N	N	%	N	%	OR (95% CI)	
Age >35 years	4629	4237	91.5	392	8.5	1.09(0.98–1.22)	0.124
Obese pre-pregnancy	3965	3374	85.1	591	14.9	2.27(2.06–2.50	<.0001
Overweight pre-pregnancy	7667	6852	89.4	815	10.6	1.51(1.39–1.64)	<.0001
Obesity at end of pregnancy	6951	5902	84.9	1049	15.1	2.68(2.47–2.90)	<.0001
Overweight at end of pregnancy	9860	8918	90.4	942	9.6	1.33(1.22–1.44)	<.0001
Normal weight at end of pregnancy	18268	17458	95.6	810	4.4	0.35(0.32–0.38)	<.0001
Excessive gestational weight gain	11422	9937	87.0	1485	13.0	2.53(2.32–2.77)	<.0001
Pregestational diabetes	304	260	85.5	44	14.5	2.01(1.46–2.77)	<.0001
Gestational diabetes	9252	8235	89.0	1017	11.0	1.60(1.48–1.73)	<.0001
Multiparity	3634	3272	90.0	362	10.0	1.33(1.18–1.49)	<.0001
Prolonged pregnancy	2086	1678	80.4	408	19.6	3.09(2.75–3.46)	<.0001
Male newborn	21802	19628	90.0	2174	10.0	1.83(1.70–1.97)	<.0001

A multiple logistic regression was performed to evaluate the independent association between the exposure variables and macrosomia (**Table 4**). The logistic regression model was statistically significant, x^2^ (9)=1021,404, p<0001.

**Table 4:** Multiple logistic regression analysis: macrosomia and significant confounding variables.

Variable	Frequency of Macrosomia (%)	Wald	aOR (95% CI)	P value
Obese pre-pregnancy	14.9	7.715	1.24 (1.07–1.44)	0.005
Obesity at end of pregnancy	15.0	102.068	2.21 (1.90–2.58)	<0.0001
Overweight at end of pregnancy	9.6	64.688	1.66 (1.46–1.87)	<0.0001
Excessive gestational weight gain	13.0	102.551	1.78 (1.59–1.98)	<0.0001
Pre-gestational diabetes	14.5	6.662	1.75 (1.15–2.69)	0.010
Gestational diabetes	11.0	39.190	1.39 (1.25–1.53)	<0.0001
Multiparity	10.0	5.623	1.24 (1.04–1.48)	0.018
Prolonged pregnancy	19.6	151.575	2.67 (2.28–3.12)	<0.0001
Male newborn	10.0	175.021	1.89 (1.72–2.08)	<0.0001

aOR, adjusted OR; Adjusted for overweight and obesity pre-pregnancy and at the end pregnancy, EGWG, pre-gestational and gestational diabetes, multiparity, prolonged pregnancy, and male newborn.

Women classified as obese pre-pregnancy (14.9% vs. 6.1%; OR 1.24 CI 1.07–1.45), overweight at the end of pregnancy (9.6% vs. 4.4%, OR 1.66; CI 1.46–1.87), and obese at the end of pregnancy (15% vs. 4.4%, OR 2.21; CI 1.90–2.58) were at increased risk for macrosomia. Obesity was an important independent predictor of macrosomia (OR 1.24), even higher if the women were obese at the end of pregnancy (OR 2.2) compared to women without obesity. Maternal overweight before pregnancy was not significantly associated with macrosomia, once adjusted for the other risks factors (OR 0.97; CI 0.85–1.11).

Macrosomia in newborns from mothers with EGWG was 13%, compared with 5.6% in those with normal weight gain. The odds of developing macrosomia among women with EGWG was 1.78 (CI 1.59–1.98).

Removing overweight at the end of pregnancy from the model decreased the prediction for obesity at the end of pregnancy (OR from 2.21 to 1.57) but increased the odds of macrosomia for women with excessive gestational weight gain (form 1.78 to 2.12).

Removing obesity at the end of pregnancy from the model decreased the prediction for overweight at the end of pregnancy (OR from 1.66 to 1.19) but increased the odds of macrosomia for women with obesity pre-pregnancy (OR from 1.24 to 1.95) and excessive gestational weight gain (form 1.17 to 2.31).

Macrosomia was present in 14.5%, 11%, and 7% of newborns from mothers diagnosed with PGD, GD, and normal glucose status, respectively. Diabetes was an independent predictor of macrosomia, with a 1.7-fold increased risk of macrosomia among women with PGD, and of 1.4-fold in women with GD.

Other risk factors for macrosomina included women with prolonged pregnancy (OR 2.67; CI 2.28–3.12) and multiparity (OR 1.24; CI 1.04–1.48). Compared with gestational age less than 39 weeks there was a 2.7-fold increase in macrosomia when gestation was beyond 41 weeks.

## Discussion

In this large Uruguayan cohort of pregnant women obesity, excessive gestational weight gain and diabetes increased the risk of macrosomia in the newborn. Moreover, this risk was significantly increased in overweight women at the end of pregnancy. These variables were independent predictors of macrosomia when adjusted for known risk factors. In this study, one third of women had BMI ≥25kg/m^2^ before pregnancy. In addition, almost half were overweight or obese at the end of it.

Overweigh and obesity pre-pregnancy was frequent in our cohort similar to that reported in other studies in Uruguay (12). For example, in a national survey of risk factors for noncommunicable diseases performed in 2013, 27.1% of women between 15 and 24 years were overweight and 12.0% obese. In addition, 32.7% women between 25 and 64 years were overweight and 29.2% obese (22).

Several studies have reported maternal obesity as an independent risk factor for newborn macrosomia (17, 23–25). Pre-gestational BMI is a documented factor that affects fetal growth (26–28). In our study obese women before pregnancy were more likely to deliver marcrosomic offspring. Koyanagi et al. in a survey in 23 developing countries in Africa, Asia, and Latin America reported that BMI significantly increased the risk of macrosomia (29).

Maternal pre-pregnancy obesity may contribute to macrosomia due to increased insulin resistance, which leads to enhanced hepatic glucose production and cause high fetal glucose and insulin concentrations (30). Fleten et al. found a direct association between pre-pregnancy BMI and birthweight in 43,705 Norwegian women, with a 20.3-g increase birthweight for a one-unit increase of BMI (31). In addition, a systematic review and meta-analysis of 31 studies with 1,443,499 women showed that maternal obesity is associated with fetal overgrowth with an increased odds of 117% for delivering a newborn with a birthweight ≥4000 g (32). In addition, in a cohort of 51,420 Uruguayan women that delivered between 2010-2012 those who where overweight or obese at the beginning and at the end of pregnancy gave birth to significantly more newborns that weighted >4250 g (33). However, few studies come from countries in South America (28, 29, 32, 34–37).

In some studies overweight women before pregnancy did not have this increased risk after adjusting for gestational weight gain (27, 38). In our study overweight women did not have an increased risk of macrosomia after adjusting for obesity at the end of pregnancy and excessive gestational weight gain. Excessive gestational weight gain is a well-recognized risk factor of macrosomia, independent of pre-pregnancy BMI (27, 39). Although overweight and obese women have the greatest prevalence of EGWG, also those with normal BMI have excess morbidity when gestational weight gain is excessive. Swank et al. reported that excessive BMI changes increased the risk of macrosomia with normal weight women having the greatest odds for macrosomia (aOR 3.85) (40). In addition, in a cohort of 23,832 Uruguayan women those with the highest changes in pregnancy weight had the greatest risk for macrosomia (3).

In our study EGWG increased 1.77 times the risk of macrosomia. However, we did not analyzed risk of macrosomia in women with EGWG according to pre-pregnancy BMI. Moreover, EGWG may have longer-term consequences in offspring. It has been associated with greater offspring BMI and higher systolic blood pressure in early adulthood as well as obesity in the first decade of life (41, 42). Hillier et al. found that the attributable risk for childhood obesity was 16.4% for excessive gestational weight gain (41). In addition, Sridhar el al. reported that excessive gestational weight gain was associated with 46% increase in odds of having overweight/obesity at 2–5 years, independent of gestational diabetes (43). In our cohort almost half women had EGWG. In an analysis of gestational weight gain of 30% of births in the United States from 2010–2011, the found that 47.2% had EGWG (44). Equally important, obese and overweight women had the uppermost excessive gestational weight gain.

Prevalence of gestational diabetes in this cohort was 22%, higher than the estimated 17% with new diagnostic IADPSG criteria adopted in 2010. Moreover, we want to highlight that this prevalence is underestimated, as data on 2-h glucose post OGTT is not available in the SIP records. A review of the global prevalence of GD between 2005 and 2015 revealed the highest prevalence in the Middle East and North Africa with a median estimate of 12.9%, followed by Southeast Asia (11.7%), Western Pacific (11.7%), South and Central America (11.2%), Africa (8.9%), and North America and Caribbean (7%). Europe had the lowermost prevalence with a median of 5.8% (45). An update of the latter review including studies from 2015 to 2018 showed a median prevalence of 15.2% in Middle East and North Africa, 15.0% in Southeast Asia, 10.3% in Western Pacific, 11.2% in South and Central America, 7.0% in North America and the Caribbean, and 6.1% in Europe (46). However, these estimates comparing different countries between 2005 and 2018 used varied screening and diagnostic values, as IADPSG criteria was proposed in 2010 and adopted by the WHO in 2013 and it is not universally accepted. The only reported prevalence in Uruguay is from a survey of 47 countries to assess its frequency globally published in 2012, which reported a 4.2% of GD (47). To our knowledge, no other data on prevalence of GD in Uruguay has been published.

Prevalence of GD has been reported to be increasing over time in different countries, probable due to increase in obesity and maternal age (48, 49). Obesity prevalence is increasing in Uruguay reflecting a growing trends worldwide (22, 50, 51). Prevalence of gestational diabetes varies by pre-pregnancy BMI, ranging from 3.6% among normal weight women (BMI 18.5–24.9) to 8.8% for class I and 13.9% for class III obesity (52). In our cohort almost 1/3 of patients had BMI ≥25 kg/m2 before pregnancy, and almost half where overweight or obese at the end of it.

Furthermore, using lower fasting glucose level thresholds supported by IADSPS identifies 6 to 11-fold more women with GD as compared to previous diagnostic criteria (53). Prevalence of PGD was similar to that reported in the literature (49). In Uruguay, in women between 15–24 and 25–64 years, diabetes has been reported in 0.4% and 7.8% of them, respectively (22). In addition, in a review of 74,420 people seeking the health card of the department of Preventive Medicine in Uruguay diabetes was reported in 1.5% of women between 15 and 19 years, as well as in 0.9% and 2.3% of those between 20–29 and 30–39 years of age, respectively (54).

As reported in other studies, PGD and GD where associated with higher prevalence and increased risk of macrosomia. Gestational diabetes has been reported to be an independent risk factor for macrosomia in a meta-analysis (55). As in our study, pregestational diabetes carried higher risk of macrosomia compared to gestational diabetes, as fasting plasma glucose is elevated from early to late pregnancy (56). Maternal hyperglycemia leads to fetal hyperglycemia and pancreatic beta cell hyperplasia and hyperinsulinism, with excess accumulation of fat and accelerated fetal growth (57). In the HAPO study there was an association between increasing maternal glucose with excessive neonatal adiposity (58).

Obesity is a well-recognized risk factor for PGD and GD. Diabetes as well as increased maternal BMI are recognized risk factors for macrosomia, as well as for overweight/obesity at a young age and type 2 diabetes in the offspring (41, 59). This may be a vicious cycle as these obese offspring may propagate an abnormal metabolic environment in utero during gestation (60, 61). Hillier et al. suggested that an overfed state created by excess glucose or overall calories may imprint the child for an overfed metabolism (41). A developmental programming may affect subsequent generations with a generational transfer of obesity, still in the lack of constant environmental stressors (62). Therefore, it may perpetuate a cycle of metabolic disorders and obesity.

On the contrary, women with GD that reduce their intake or have placental dysfunction due to hyperglycemia can have the opposite effect with gestational undernutrition and future metabolic and cardiovascular programming in postnatal and adult life. This adverse development in utero revealed by low birth weight has been associated with greater risk of cardiovascular disease, lung disease, and polycystic ovary syndrome. In addition, timing of this undernutrition may program susceptibility to disease in different ways. When nutrient restriction occurs in early gestation, it has been associated with adult hypertension, but if ensues during late gestation, it has been related to impaired glucose tolerance, type 2 diabetes, and visceral adiposity (63, 64).

Lifestyle interventions directed towards healthy eating and physical activity can have enormous impact on maternal health as well as reduce childhood obesity. Reducing the prevalence of overweight and obesity amid women of reproductive age could reduce macrosomia and gestational diabetes. Preconception care guidelines recommend screening women with annual BMI and counseling and referral accordingly (65). Interventions to prevent excess gestational weight gain during pregnancy may be more feasible as women are more motivated to improve their health behavior (66). However, research about the interventions to improve gestational weight gain has not shown a significant change in perinatal outcomes such as macrosomia (67, 68). More research is needed to clarify this point and to evaluate these interventions in the long-term in mothers and offspring.

In addition, treatment of gestational diabetes with diet or insulin was associated with lower risk of macrosomia in a meta-analysis of four randomized controlled trials from developed countries (69). Likewise, glycemic control during pregnancy in pregestational diabetes reduces the risk of macrosomia (70).

Additional recognized risk factors for macrosomia include prolonged pregnancy, multiparity, and male newborn. It has been reported that prolonged pregnancy beyond the expected delivery day is a risk factor for macrosomia, as fetuses gain approximately 150–200 g weekly near term (29, 71, 72). Advanced gestational age ends in a greater birth weight, with macrosomia accounting for 3%–10% of post-term deliveries (73).

Multiparity has been associated with macrosomia maybe due to the fact that birth weight increases with parity, with rates of macrosomia 2–3 times higher than women without this risk factor. There is evidence that with every newborn the weight increase 100 to 150 g (29, 74). Male gender has been associated with increased odds of macrosomia, as male newborns weight more than females at any gestational age (75).

The increased risk of fetal macrosomia and maternal ages is well established (76), related to metabolic changes associated to age (77). We did not find an association between older maternal age and macrosomia. However, we did not consider gestational age at delivery as older maternal women may have delivered at an earlier gestation age decreasing the probability of attaining a >4 kg neonate (77).

There are some limitations in our study. First, in the SIP records the information about the 2-h glucose after an oral glucose tolerance test (OGTT) is not available. Using only fasting blood glucose as diagnostic criteria will miss approximately 5%–15% patients with GDM (78). This would lead to an underestimation of the prevalence of GD as well as it effect on the risk of macrosomia. Second, there was no data on glucose control or use of insulin on patients with diabetes, which could affect gestational weight gain. In addition, it may influence macrosomia as appropriate management of GD with nutritional therapy and insulin if not well controlled with diet alone has been reported to reduce its rates (79). Third, pre-pregnancy weight was self-reported, which may have errors in BMI and gestational weight gain. However, self-reported pre-pregnancy weight has been shown to highly correlate with measured weight with average error being small (80). However, women with higher BMI and minority groups are more likely to misreport weight, with obese women being more likely to underestimate their weight. Fourth, no information on physical activity or diet is available in the SIP form, which may impact gestational weight gain. Finally, socio-demographic data included in the SIP form (ethnic group, education level and marital status) were not recorded in the majority of cases, so these could not be included in the analysis. As few studies come from Latin America, with reported differences in population characteristic from the other regions where most of the findings in this issue have been published, this information could have been helpful to assess these differences.

We believe this study contributes to the field as few studies about excessive maternal weight before or during pregnancy and risk of macrosomia are coming from South America. Most studies come form Western Europe and North America, with differences in population socioeconomics, ethnics, and educational status, making the results of this study of great relevance. This research highlights the importance of weight management amid women of reproductive age in this region. In addition, there are scant data about diabetes during pregnancy in Latin America, with this study contributing in showing a high occurrence of gestational diabetes as well as its role as a risk factor for macrosomia.

## Conclusion

Maternal overweight, obesity, EGWG, and GD are prevalent in Uruguay, increasing the risk macrosomia. Overweight/obesity are recognized risk factors of various other complications such as PGD, GD, and long-term health consequences on offspring health and wellbeing. We believe that efforts to implement strategies to decrease the prevalence of obesity and overweight among women of reproductive age are essential to improve maternal and neonatal outcomes.

## Data Availability Statement

The raw data supporting the conclusions of this article will be made available by the authors, without undue reservation.

## Ethics Statement

Ethical review and approval was not required for the study on human participants in accordance with the local legislation and institutional requirements. Written informed consent for participation was not required for this study in accordance with the national legislation and the institutional requirements.

## Author Contributions

Wrote the first draft of the manuscript: MP. Contributed to the writing of the manuscript: JP. Made contributions to the acquisition of the clinical data: JP and IB. Agreed with manuscript results and conclusions: JP, IB, and MP. All authors contributed to the article and approved the submitted version.

## Conflict of Interest

The authors declare that the research was conducted in the absence of any commercial or financial relationships that could be construed as a potential conflict of interest.
